# Extended carrier lifetimes and diffusion in hybrid perovskites revealed by Hall effect and photoconductivity measurements

**DOI:** 10.1038/ncomms12253

**Published:** 2016-08-01

**Authors:** Y. Chen, H. T. Yi, X. Wu, R. Haroldson, Y. N. Gartstein, Y. I. Rodionov, K. S. Tikhonov, A. Zakhidov, X. -Y. Zhu, V. Podzorov

**Affiliations:** 1 Department of Physics, Rutgers University, Piscataway, New Jersey 08854, USA; 2 Department of Chemistry, Columbia University, New York, New York 10027, USA; 3 Department of Physics and NanoTech Institute, University of Texas at Dallas, Richardson, Texas 75080, USA; 4 The Institute for Theoretical and Applied Electrodynamics, The National University of Science and Technology, MISIS, Moscow 119049, Russia; 5 Landau Institute for Theoretical Physics, Moscow 119334, Russia; 6 Institute for Adv. Mater. and Devices for Nanotech., Rutgers University, Piscataway, New Jersey 08854, USA

## Abstract

Impressive performance of hybrid perovskite solar cells reported in recent years still awaits a comprehensive understanding of its microscopic origins. In this work, the intrinsic Hall mobility and photocarrier recombination coefficient are directly measured in these materials in steady-state transport studies. The results show that electron-hole recombination and carrier trapping rates in hybrid perovskites are very low. The bimolecular recombination coefficient (10^−11^ to 10^−10^ cm^3^ s^−1^) is found to be on par with that in the best direct-band inorganic semiconductors, even though the intrinsic Hall mobility in hybrid perovskites is considerably lower (up to 60 cm^2^ V^−1^ s^−1^). Measured here, steady-state carrier lifetimes (of up to 3 ms) and diffusion lengths (as long as 650 μm) are significantly longer than those in high-purity crystalline inorganic semiconductors. We suggest that these experimental findings are consistent with the polaronic nature of charge carriers, resulting from an interaction of charges with methylammonium dipoles.

## Abstract (web summary)

Hybrid perovskites exhibit long carrier diffusion lengths and lifetimes. Here, Chen *et al*. show experimentally that carrier recombination in perovskites is far from Langevin and closer to the best direct-bandgap semiconductors, which can be explained by the dipolar polaronic nature of charge carriers.

## Graphical abstract

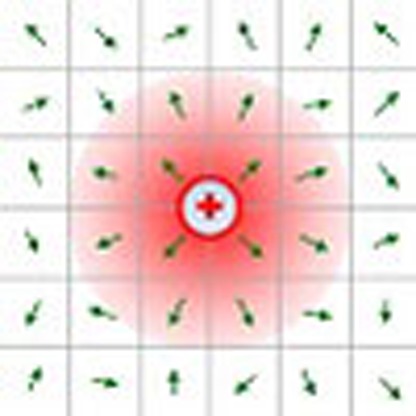

Hybrid (organic–inorganic) perovskite solar cells represent the recent breakthrough in photovoltaic applications with reported power conversion efficiencies reaching 20% (refs 1, 2, 3). In addition to the abundance of the applied studies on this topic, there is a great interest in understanding the fundamental transport and photophysical properties of these materials, the picture of which is not firmly established yet, thereby calling for more reliable experimental studies. For instance, a possibility of high charge-carrier mobility has been considered as one important factor that contributes to the excellent photovoltaic performance of hybrid lead-halide perovskites. Yet, an unambiguous determination of the intrinsic mobility in these materials is missing. Existing experimental values in similar materials range from 0.6 to 50 cm^2^ V^−1^ s^−1^ (refs 4, 5, 6). However, most of the important transport and photophysical parameters, including the carrier mobilities, lifetimes and recombination rates, were so far determined either in materials different from those relevant for high-performance solar cells (for instance, in metallic tin-halide instead of insulating lead-halide perovskites, or perovskites that adopt 2D layered rather than 3D cubic structure) or obtained indirectly, under the conditions less relevant to applications (for instance, in ultrafast spectroscopic experiments, rather than steady-state transport measurements).

Here, we report artefact-corrected Hall effect and steady-state photoconductivity measurements carried out in a range of thin films and single crystals of exemplary hybrid perovskites, CH_3_NH_3_PbI_3_ and CH_3_NH_3_PbBr_3_, of current interest for photovoltaic applications. Hall effect allows us to directly and independently address the density of photogenerated carriers, *n*_Hall_, and the intrinsic carrier mobility, *μ*_Hall_, without assumptions typical for other methods, such as in ultrafast spectroscopic techniques or space-charge-limited current measurements. We find that, in a wide range of illumination intensities, the dynamics of photocarriers is governed by bimolecular electron-hole (e-h) recombination with a very small recombination coefficient *γ* in the range of 10^−11^ to 10^−10^ cm^3^ s^−1^, which is comparable to the values observed in the best single-crystalline direct-band inorganic semiconductors, such as GaAs, even though the measured intrinsic Hall mobilities are moderate (*μ*_Hall_ of up to 60 cm^2^ V^−1^ s^−1^ in perovskite single crystals) and smaller than *μ* in typical inorganic semiconductors by 1–3 orders of magnitude. In addition, the carrier lifetime, *τ*, and diffusion length, *l*, directly measured in our steady-state transport experiments are found to be remarkably long (*τ* is up to 30 μs and *l* is up to 23 μm in polycrystalline films, and up to 3 ms and 650 μm in single crystals, respectively). Our experiment thus provides a direct steady-state measurement quantitatively revealing a low-rate photocarrier recombination and negligible trapping, as well as extremely long carrier lifetimes and diffusion lengths in hybrid perovskites. While in agreement with some of the recent theoretical predictions^7^,^8^,^9^,^10^, these results accentuate important questions as of the physical origins of the found intrinsic carrier mobility, e-h recombination and trapping rates in these materials synthesized via inexpensive vapour- or solution-based routes at temperatures close to room temperature. We propose rationalization of some of our findings based on the picture of re-organization of the methylammonium dipoles around the charge carriers. This interaction leads to carrier relaxation into polarons, whose properties differ from the bare band carriers^11^,^12^,^13^.

## Results

### a.c. Hall effect measurements of charge-carrier mobility

It should be emphasized that unambiguous determination of the intrinsic (that is, not dominated by traps) charge-carrier mobility requires precise Hall effect measurements, which are quite challenging in highly resistive materials with relatively low *μ*, such as organic semiconductors or the hybrid perovskites studied here (see, for example, ref. 14). The major challenges are associated with a very high resistivity of pure stoichiometric perovskites (greater than GΩ), related to the negligible (in the dark) density of charge carriers, and a poor signal-to-noise ratio in conventional d.c. Hall measurements of low-*μ* materials. To overcome these difficulties, here, we have developed a specialized highly sensitive a.c. Hall measurement technique, corrected for the Faraday-induction artefacts, in which a low-frequency a.c. magnetic field, **B**, is applied perpendicular to the sample's surface, while a d.c. current, *I*, is passed through the sample, and an a.c. Hall voltage, *V*_Hall_, is detected across the channel by a phase-sensitive lock-in technique, which allows to markedly increase the signal-to-noise ratio^15^. A parasitic Faraday-induction electro-motive force, occurring in a.c. Hall measurements at the same frequency as *V*_Hall_, is usually comparable to the actual Hall voltage signal and can easily compromise these measurements. Therefore, Faraday-induction-corrected a.c. Hall measurements, as implemented here in perovskites, are absolutely necessary to obtain reliable data (see the ‘Methods' section, Supplementary Fig. 1 and Supplementary Note 1)^15^. Pure CH_3_NH_3_PbI_3_ samples are highly resistive in the dark (typical *R*⩾100 GΩ), and thus we utilize a steady-state monochromatic photo-excitation (*λ*=465 nm) to generate a population of carriers and be able to measure Hall effect. In CH_3_NH_3_PbBr_3_ single crystals, we were able to measure a Hall effect in the dark as well, because these crystals are weakly conducting in the dark (at a level of 50 MΩ). We perform all our measurements in a 4-probe/Hall bar geometry to account for contact-resistance effects and ensure that channel conductivity, *σ*, as well as the Hall mobility and carrier density, *μ*_Hall_ and *n*_Hall_, are determined correctly.

### Steady-state photoconductivity vs. light intensity

**Figure 1:**
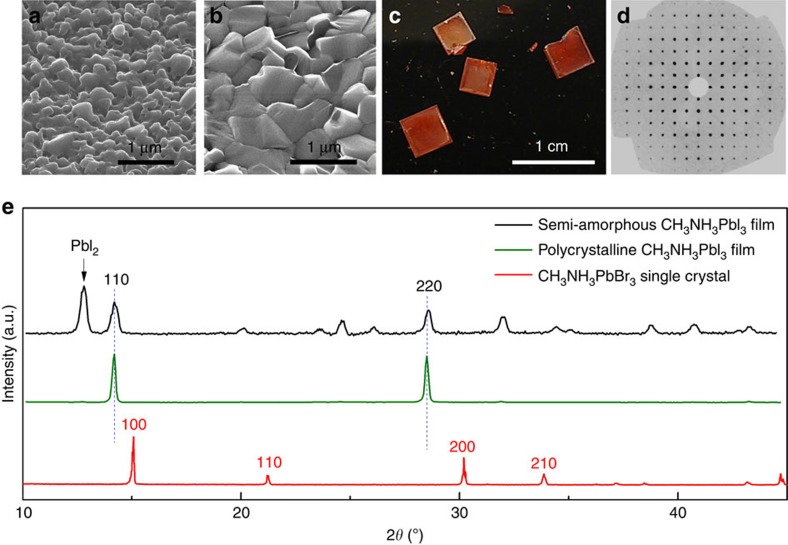
Morphological and structural characterization of hybrid perovskites used in this study. (**a**,**b**) Helium-ion microscope images of CH_3_NH_3_PbI_3_ thin films grown by solution and vapour methods, respectively (see the ‘Methods' section). Vapour-grown films clearly show much higher crystallinity. (**c**) Optical photograph of solution-grown CH_3_NH_3_PbBr_3_ single crystals. (**d**) A reconstructed precession image of the hk0 level from single-crystal X-ray diffraction of these single crystals, showing perfect cubic crystalline structure with low density of defects. (**e**) Typical powder X-ray diffraction curves of all three types of samples shown in **a**–**c**, semi amorphous film (black), polycrystalline film (green) and single crystals (red). The arrow indicates the peak corresponding to unreacted PbI_2_ precursor, and vertical dashed lines show the (110) and (220) peaks of the fully stoichiometric CH_3_NH_3_PbI_3_.

Three types of hybrid perovskite samples were used in our study (Fig. 1). (a) Semi amorphous, solution-grown thin films, (b) polycrystalline, vapour-grown thin films and (c) highly ordered, solution-grown single crystals. The microscopy and X-ray diffraction clearly show that the vapour-processed films have a much better crystallinity than the solution-processed ones, and our single crystals have excellent quality (see Fig. 1 and see the ‘Methods' section for details).

**Figure 2:**
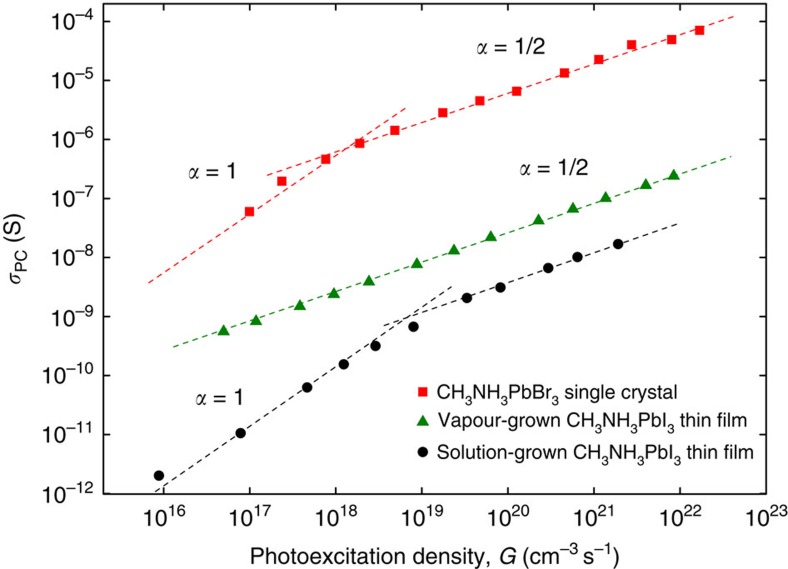
Steady-state photoconductivity measured as a function of photo-excitation density in thin films and single crystals of hybrid perovskites. Measurements are carried out by 4-probe technique under a cw photo-excitation with a blue light (*λ*=465 nm) in CH_3_NH_3_PbBr_3_ single crystals (red squares), vapour-grown polycrystalline CH_3_NH_3_PbI_3_ films (green triangles) and solution-grown semi amorphous CH_3_NH_3_PbI_3_ films (black circles). Dashed lines are the power law fits, *σ*_PC_∝*G*^*α*^, with the exponents *α*=1 or ½ (as indicated). It is clear that bimolecular e–h recombination (*α*=½) dominates the behaviour at high photo-excitation densities (note the double-log scale).

We have found that all our high-quality (stoichiometric) samples exhibit a very low dark conductivity. Nevertheless, a significant photoconductivity is observed in all of them. Figure 2 shows typical dependences of a steady-state photoconductivity, *σ*_PC_, on photo-excitation density, *G*, which always follows a power law, *σ*_PC_∝*G*^*α*^, with the exponent *α*=1 or ½ (linear or square-root regimes). Photo-excitation density, *G*, is defined as the incident photon flux *F* (in cm^−2^ s^−1^) divided by the absorption length of the material.

The observed *σ*_PC_(*G*) dependence can be understood in terms of charge-carrier monomolecular decay (trapping in the linear regime) or bimolecular decay (e-h recombination in the square-root regime). The photocarrier density, *n* (*n*≡*n*_e_≈*n*_h_), in steady-state measurements is found from the following rate equation, with d*n*/d*t*=0 at dynamic equilibrium (see, for example,^16^):


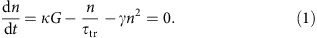


Here, *κG* is the rate of carrier generation via photon absorption resulting in production of free electrons and holes with probability *κ* per photon (the photocarrier-generation efficiency). The second and third terms represent the two channels of carrier decay: the trapping and e-h recombination, where *τ*_tr_ is the trap-limited carrier lifetime (an average time carriers diffuse before being trapped), and *γ* is the coefficient of e-h recombination. In the carrier density range probed here, we do not see any experimental evidence of the third-order (Auger) processes, which are therefore excluded from equation (1). At low photo-excitation intensities, when the concentration of electrons and holes is small, the dominant process limiting the carrier lifetime is trapping, and *γn*^2^ term in equation (1) can be neglected, leading to a linear regime in photoconductivity: *σ*_PC_≡*eμn*=*eμκτ*_tr_*G*, where *e* is the elementary charge. With increasing excitation intensity, the bimolecular recombination eventually becomes dominant, resulting in a transition from the linear to a sublinear regime: *σ*_PC_=*eμ*·(*κ*/*γ*)^1/2^·*G*^1/2^, obtained from equation (1) by neglecting the *n*/*τ*_tr_ term. Figure 2 shows that these two regimes are indeed what is observed in hybrid perovskites. Highly crystalline samples exhibit bimolecular recombination regime (*α*=1/2) in a wider range of excitation intensities, which is consistent with the higher *σ*_PC_ and *μ*_Hall_ in the crystal samples (see below).

### Hall measurements under photo-excitation and in the dark

**Figure 3:**
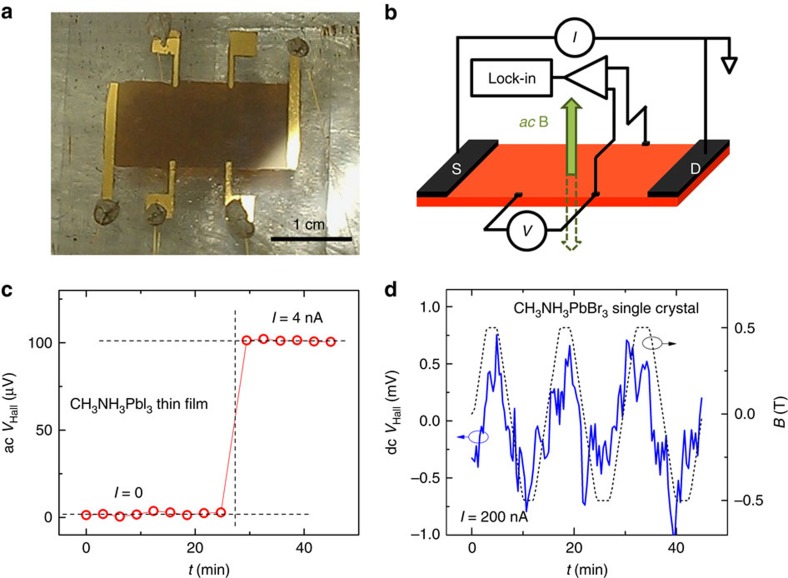
Hall effect measurements in hybrid lead-halide perovskites. (**a**) A photo of typical solution-grown CH_3_NH_3_PbI_3_ thin film on glass with Au contacts in a 4-probe/Hall bar geometry. (**b**) A diagram of the a.c. Hall effect measurement setup (see description in text and ref. 15). (**c**) A representative Faraday-induction-corrected a.c. Hall measurement in a solution-grown CH_3_NH_3_PbI_3_ thin film shown in **a**. When a d.c. excitation current of 4 nA is driven through the film uniformly illuminated with a blue light (*λ*=465 nm) and subjected to an a.c. *B* field of r.m.s. magnitude 0.23 T at 0.5 Hz, an a.c. Hall voltage of 100 μV is detected above the zero-bias background by using a lock-in technique shown in **b**. The vertical and horizontal dashed lines show the moment d.c. excitation current is turned on (at *t*=28 min) and the two levels of the Hall voltage, at *I*=0 and 4 nA, respectively. Note that even though Hall mobility of this sample is only *μ*_Hall_=1.5 cm^2^ V^−1^ s^−1^, the signal-to-noise ratio is excellent (the standard deviation in *V*_Hall_ among consecutive six measurements is only about 0.5%). (**d**) A representative d.c. Hall measurement in CH_3_NH_3_PbBr_3_ single crystals. *V*_Hall_ is measured with an electrometer at a constant excitation current *I*=200 nA, while *B* field is slowly swept between −0.5 T and 0.5 T. Despite the much higher mobility of this sample (*μ*_Hall_=11±3 cm^2^ V^−1^ s^−1^), the signal-to-noise ratio is much worse, with the large error imposed by the noise.

To decouple the carrier density and mobility in *σ*_PC_=*enμ* and obtain the microscopic parameters describing the carrier dynamics, *τ*_tr_ and *γ*, from the rate equation for *n* (equation (1)) and experimental data, one needs to know the intrinsic charge-carrier mobility *μ*. For this purpose, we have performed Hall effect measurements, as discussed above, using 4-probe/Hall-bar device structures (Fig. 3a) and a variant of an a.c. Hall measurement technique (Fig. 3b) specifically adjusted for these materials (see the ‘Methods' section, Supplementary Fig. 1 and Supplementary Note 1). A typical measurement result is shown in Fig. 3c: a very clear and quiet a.c. Hall signal as detected in a solution-grown CH_3_NH_3_PbI_3_ thin film that has a Hall mobility of only *μ*_Hall_=1.5 cm^2^ V^−1^ s^−1^. Reliable Hall measurements with such an excellent signal-to-noise ratio in highly resistive systems with carrier mobilities as low as 1 cm^2^ V^−1^ s^−1^ are unprecedented. For comparison, conventional d.c. Hall measurements performed in a perovskite single crystal with a much higher mobility, *μ*_Hall_=11 cm^2^ V^−1^ s^−1^, shown in Fig. 3d, evidently exhibit a much noisier signal. It is clear that Faraday-induction-corrected a.c. Hall measurements are by far superior to the conventional d.c. technique in terms of the signal-to-noise ratio, even though it uses a smaller magnetic field (r.m.s. *B*=0.23 T).

As expected for pure undoped band insulators, our CH_3_NH_3_PbI_3_ samples are highly resistive in the dark (with a typical sample resistance >100 GΩ). Thus, we used a cw photo-excitation to generate photocarriers and perform steady-state photo a.c. Hall measurements. In a system with photogenerated electrons and holes (*n*_e_≈*n*_h_≡*n*) and a negligible concentration of dark carriers, Hall voltage is given by:


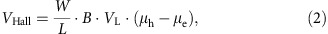


where *W* and *L* are the channel's width and length, respectively, *V*_L_ is the longitudinal voltage drop along the channel (corrected for contact effects by using the 4-probe technique), and *μ*_h_, *μ*_e_ are the mobilities of holes and electrons, respectively. Equation (2) shows that photo Hall effect measurements yield the difference between the electron and hole mobilities, rather than their absolute values. In the context of the perovskites under study, recent calculations showed that *μ*_e_ and *μ*_h_ in these materials should differ from each other by an amount comparable to the mobilities themselves^7^,^17^,^18^,^19^,^20^. Indeed, theoretical calculations have consistently indicated that while effective masses of electrons, *m*_*e*_, and holes, *m*_*h*_, have the same order of magnitude, there is also a noticeable difference between the two, ranging from 20 to 200%, depending on the specific computational method used^18^. A recent THz spectroscopy study also suggests a difference of a factor of 2 between the electron and hole mobilities^21^.

Therefore, even though precise values of *μ*_e_ and *μ*_h_ cannot be obtained from photo Hall measurements, the difference, Δ*μ*≡*μ*_h_—*μ*_e_∼*μ*, extracted from equation (2) would yield a faithful representation. In turn, the carrier density obtained from the photo Hall effect measurements is: *n*=*n*_e_+*n*_h_=*σ*_PC_/(*e*·*μ*), which is also a good approximation for the actual density of photogenerated charges. The data presented below are analysed using this association. The dark Hall effect measurements possible in weakly conducting single-crystal CH_3_NH_3_PbBr_3_ samples give further credence to the approach.

**Figure 4:**
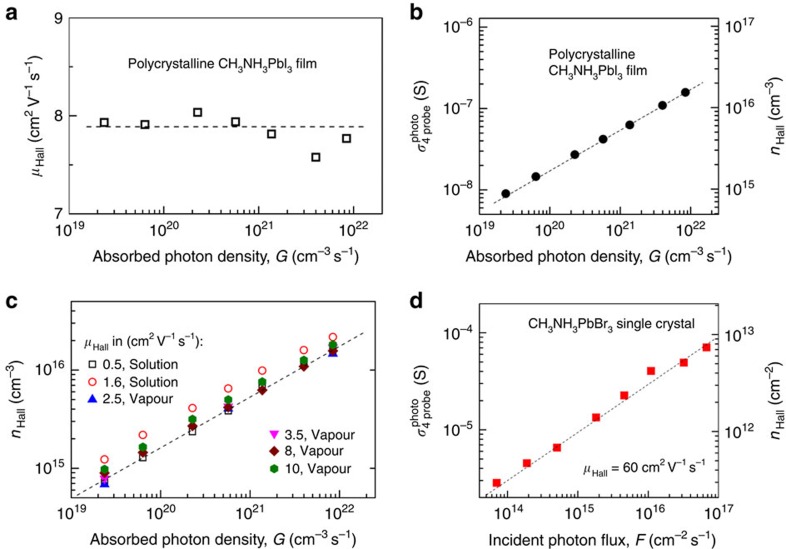
Steady-state photoconductivity and Hall effect measurements in perovskite films and crystals. (**a**) *μ*_Hall_ in a vapour-grown 100 nm-thick polycrystalline CH_3_NH_3_PbI_3_ film (similar to that shown in Fig. 1b), measured at different photo-excitation densities, is nearly a constant (the dashed line indicates an average value of≈8 cm^2^ V^−1^ s^−1^). (**b**) Photoconductivity and Hall carrier density measured in this film as a function of absorbed photon density. (**c**) *n*_Hall_(*G*) in six thin-film samples with very different morphologies and *μ*_Hall_ values, showing that bimolecular recombination (*n*_Hall_∝*G*^1/2^) governs all these samples. Hall mobility and the method of fabrication are indicated for each film in the legend. (**d**) Photoconductivity and Hall carrier density measured in a macroscopic, bulky CH_3_NH_3_PbBr_3_ single crystal (as the ones shown in Fig. 1c) with a (dark) Hall mobility *μ*_Hall_≈60 cm^2^ V^−1^ s^−1^ (for holes). Dashed lines in **b**–**d** are the power law fits with exponent *α*=½: *n* or *σ*=constant·*G*^1/2^.

Figure 4 presents Hall effect data for a variety of hybrid perovskite samples. Fig. 4a shows that Hall mobility, *μ*_Hall_=8±0.4 cm^2^ V^−1^ s^−1^, measured in a polycrystalline CH_3_NH_3_PbI_3_ film remains almost constant over the range of nearly three orders of magnitude in light intensity (the error is defined by the fluctuations in Fig. 4a). The 4-probe photoconductivity, *σ*_PC_, and the density of photogenerated charge carriers, *n*_Hall_, determined from the simultaneous longitudinal *σ*_PC_ and Hall effect measurements in these polycrystalline films are plotted in Fig. 4b. Within the entire measurement range, the carrier density dependence on the illumination intensity exhibits *α*=½ power law: *n*_Hall_∝*G*^1/2^. We emphasize that independent measurements of *μ*_Hall_ as a function of photo-excitation density are essential for obtaining *n*_Hall_(*G*) dependence and therefore for determination of the microscopic transport parameters, *τ*_tr_ and *γ*, by using equation (1) to fit the data. With the density of photogenerated carriers determined experimentally, we can now interpret their dynamics with the help of equation (1). As pointed out above, under a steady-state photo-excitation, equation (1) gives *n*=(*κ*/*γ*)^1/2^·*G*^1/2^ in the regime governed by a bimolecular recombination, that is, when the carrier trapping time *τ*_tr_ well exceeds the time of e-h recombination *τ*_r_, *τ*_tr_>>*τ*_r_≡(*γn*)^−1^. Fitting the experimental Hall carrier density in Fig. 4b with this *n*(*G*) relationship yields the upper bound for the bimolecular e-h recombination coefficient *γ* in polycrystalline perovskite films, *γ*≤3 × 10^−11^ cm^3^ s^−1^ (for the photocarrier-generation efficiency *κ*≤100%).

The measurements shown in Fig. 4 can also be used to directly estimate the steady-state charge-carrier lifetime, either *τ*_tr_ or *τ*_r_, and diffusion length, *l*. Indeed, since bimolecular recombination (*α*=½) apparently dominates in the entire range of photon densities in Fig. 4b, we must have the condition *n*/*τ*_tr_<<*γn*^2^ (or, *τ*_tr_>>*τ*_r_≡(*γn*)^−1^) fulfilled for all the incident photon intensities, including the lowest one, at which *n*_Hall_=9 × 10^14^ cm^−3^ (Fig. 4b). Therefore, the effective trap-limited carrier lifetime *τ*_tr_ in the disordered polycrystalline film in Fig. 4b must be longer than the lifetime limited by bimolecular e-h recombination, *τ*_tr_>*τ*_r_≈30 μs. This estimate shows that even in disordered CH_3_NH_3_PbI_3_ thin films, charge carriers have an extremely long lifetime, as far as trapping is concerned, and thus the effective density of the corresponding deep traps must be very low, or the traps must be electronically passivated. The corresponding lower bound of trap-limited carrier diffusion lengths in these polycrystalline perovskite films is: *l*=(*Dτ*_tr_)^1/2^∼23 μm. Here, we needed the Hall mobility again to calculate the diffusion coefficient, *D*∼*μ*_Hall_*k*_B_*T*/*e*, where *k*_B_ is the Boltzmann constant and *T*=300 K. Note that negligible trapping and a very long diffusion length, greater than the grain size in our polycrystalline films (Fig. 1b), are consistent with the recent theoretical studies predicting the presence of only shallow traps and benign grain boundaries that do not trap carriers in perovskites^9^,^10^. Of course, given the dominant e-h recombination, the actual carrier lifetime in the *α*=½ regime is a decreasing function of photo-excitation density *G*: *τ*_r_=(*γn*)^−1^ (see also^16^).

Figure 4c shows *n*_Hall_ and *μ*_Hall_ in six different solution and vapour-grown polycrystalline thin films. In general, vapour-grown samples have an appreciably higher *μ*_Hall_, consistent with their better crystallinity. In sharp contrast, *n*_Hall_(*G*) does not seem to correlate much with the preparation method and mobility. Indeed, while *μ*_Hall_ differs among the six samples by as much as a factor of 20, *n*_Hall_ varies only within a factor of two. Correspondingly, *γ* values are also very similar for all these samples, *γ*∼(1–5) × 10^−11^ cm^3^ s^−1^. This suggests that while film morphology has a clear effect on charge transport, it has little effect on photocarrier generation and recombination. This is consistent with the notion that bimolecular recombination in this system is not governed by carrier diffusion, therefore we do not see a strong correlation between the recombination dynamics and charge-carrier mobility.

Finally, we have performed Hall measurements in CH_3_NH_3_PbBr_3_ single crystals (Fig. 4d). One important difference in this case is that these crystals are weakly conducting in the dark, which allows us to perform Hall effect measurements in the dark and obtain the mobility of holes (*μ*_Hall_=60±5 cm^2^ V^−1^ s^−1^), without having an ambiguity of charge compensation as in photo Hall measurements. Photoconductivity in these crystals is much higher than that in thin films and also exhibits a well-defined *α*=½ behaviour (Fig. 4d). A similar analysis of *n*_Hall_(*G*) dependence shows that the e-h recombination coefficient in these crystals is *γ*∼8 × 10^−11^ cm^3^ s^−1^ (for more details on the extraction procedure see Supplementary Note 2). By defining the effective recombination-limited carrier lifetime again as *τ*_r_=(*γn*)^−1^ and using the experimental carrier density from Hall effect measurements, we can determine the carrier lifetime and diffusion length in these single crystals. At the lowest incident photo-excitation flux in Fig. 4d, corresponding to the measured projected carrier density *n*_Hall_=3 × 10^11^ cm^−2^ and the effective bulk carrier density *n*∼4.6 × 10^12^ cm^−3^ (Supplementary Note 2), we find: *τ*_r_∼2.7 ms, and *l*≡(*Dτ*_r_)^1/2^∼650 μm, which are remarkably long for a solution-grown semiconductor. We emphasize that these values represent the lower limit for the trap-limited carrier lifetime, *τ*_tr_, and diffusion length, *l*_tr_, since the condition *τ*_tr_>>*τ*_r_ must be satisfied in the entire regime dominated by a bimolecular recombination, thus indicating again that trapping is strongly suppressed in these materials. We must add that *σ*_PC_(*G*) in analogous lead iodide (CH_3_NH_3_PbI_3_) single crystals (not shown here) are qualitatively similar, except that these crystals are highly insulating in the dark, and thus only photo Hall measurements were possible, yielding an estimate for *μ*∼few cm^2^ V^−1^ s^−1^.

### Theoretical estimates of e-h recombination coefficients

E-h recombination in semiconductors is a fundamentally important process, and various approaches have been developed for assessing the corresponding kinetic coefficient *γ*. In one approach, for instance, *γ* is associated with the product *sv* of the Coulomb capture cross section *s* and carrier thermal velocity *v* (ref. 16). In the case of disordered organic and inorganic semiconductors, recombination of charge carriers is often described by the Langevin model^22^,^23^, which leads to 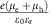 for the recombination coefficient, where *ɛ*_0_ and *ɛ*_r_ are the dielectric permittivities of vacuum and the material, respectively. Evaluating these expressions for our systems would yield estimates of *γ* on the order of 10^−6^ cm^3^ s^−1^ or higher, that is 4–5 orders of magnitudes greater than what we find experimentally. One, of course, realizes that the above models, evidently not applicable to our case, refer to the e-h collision events rather than to the radiative recombination *per se*. A more appropriate approach that was successfully applied to the actual radiative recombination in inorganic semiconductors is based on the van Roosbroeck–Shockley's theory rooted in the principle of detailed balance (for reviews, see, for instance, refs 24, 25). This theory, in particular, explains well the values of *γ*∼10^−10^ cm^3^ s^−1^ exhibited by high-purity direct-band inorganic semiconductors, such as, for instance, GaAs^26^,^27^ (see also http://www.ioffe.ru/SVA/NSM/Semicond/index.html), which, remarkably, are comparable to the values we extract from our observations in hybrid perovskites. In the van Roosbroeck–Shockley's approach, the radiative recombination coefficient *γ* is established by the system's properties in the thermal equilibrium (that is, in the dark):


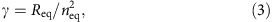


where, *R*_eq_ and *n*_eq_ are the equilibrium (dark) recombination rate and concentration of electrons (equal to that of holes), respectively. Furthermore, *R*_eq_ is related thermodynamically to the optical properties of the system as^25^:


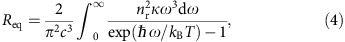


utilizing the frequency *ω*-dependent refraction, *n*_r_(*ω*), and extinction, *κ*(*ω*), coefficients. An assessment of equation (3) can be made using model considerations (as illustrated in Supplementary Note 3). Even more attractively, one can use the actual experimental optical data to evaluate *R*_eq_ in equation (4), for which here we use the optical parameters (see Supplementary Fig. 2 and Supplementary Note 3) of CH_3_NH_3_PbI_3_ perovskite extracted from accurate ellipsometric measurements in ref. 28. On the other hand, the dark carrier concentration at the thermal equilibrium *n*_eq_ is not measured directly. If one were to use the standard textbook expression for a non-degenerate semiconductor with energy gap *E*_g_ separating two parabolic bands^27^:


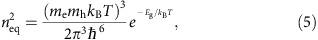


equation (3) then yields the recombination coefficient:


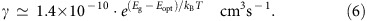


**Figure 5:**
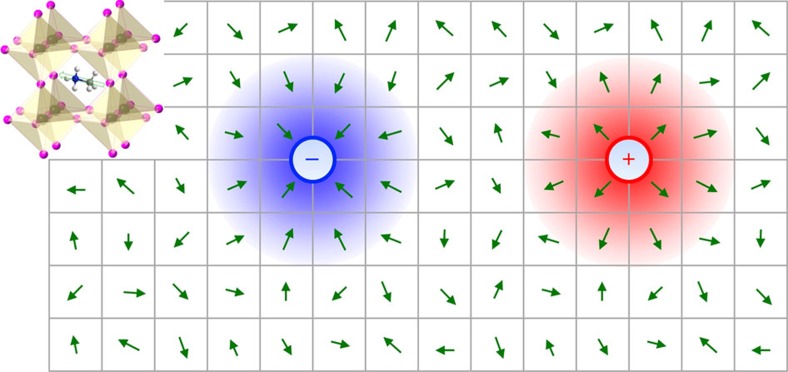
Dipolar polarons in hybrid perovskite lattice. Schematically, charge carriers can induce rotational re-organization of the surrounding CH_3_NH_3_ dipoles (green arrows), leading to the formation of dipolar polarons. Such polarons could account for the relatively low intrinsic charge-carrier mobilities and reduced bimolecular recombination coefficients experimentally revealed in this work. The structure of hybrid perovskite unit cell, with PbI_6_ or PbBr_6_ octahedra and a polar methylammonium molecular cation at the centre, is shown in the upper left corner. The green arrows in the main panel represent these polar methylammonium cations.

Here, the prefactor was calculated with *T*=300 K and *m*_*e*_≃*m*_*h*_=*m*=0.2*m*_0_ (*m*_0_ being the free electron mass). Equation (6) features the absorption onset parameter denoted *E*_opt_, which is equal to 1.553 eV in the parameterization of ref. 28. The excitonic (e-h attraction) effects^11^,^25^ are known to reduce the onset of optical absorption in comparison with the semiconductor band-gap in perovskites (see, for example, the discussion of excitons in ref. 29). One can approximately assess the resulting exponential factor in equation (6) from the corresponding exciton binding energy *E*_X_. For *m*=0.2*m*_0_ and the relative permittivity equal to 5, for instance, *E*_X_≃54 meV, indicating that the exponential factor is of the order of 10, which would make result (6) for *γ* larger than our experimental values by about one order of magnitude. The discrepancy here might result from the underestimate of *n*_eq_ by equation (5) for the conventional band carriers, and the larger values of *n*_eq_ would lead to a better agreement with the experiment. We suggest that larger concentrations *n*_eq_ might be actually realized in perovskites due to the interaction of band charge carriers with methylammonium dipoles, as illustrated in Fig. 5.

### Accounting for polaronic effects

Theoretical calculations by Frost *et al*.^7^ demonstrate that the methylammonium dipoles in hybrid perovskites carry a significant dipole moment, *P*=2.29 D, and create a rough potential landscape at the nanoscale, but can be easily rotated or locally aligned by overcoming a small rotational energy barrier, *U*_rot_≈1 kJ mol^−1^ (1.6 × 10^−21^ J or 10 meV per dipole). Here, we propose that a charge carrier moving through the perovskite lattice may itself induce a local orientational rearrangement of the surrounding methylammonium dipoles tending to align them along its electric field (Fig. 5), thus resulting in a type of a dipolar polaron, conceptually similar to polarons known in ionic crystals and polar semiconductors^11^,^12^,^13^. The estimates outlined in Supplementary Note 3 show that such polarons in perovskites should be characterized as intermediate-coupling polarons^12^, with the dimensionless electron–phonon coupling constant *α*_*e−ph*_≃2.5 being a good representative value for the interaction of the band carriers with the longitudinal dipolar vibrational modes of energy *ħω*_0_∼10 meV. The ‘dressing' of a band carrier by a phonon cloud is known to change its properties^11^,^12^,^13^: the standard band carrier dispersion *E*(*k*)=*ħ*^2^*k*^2^/2*m* would be modified to the polaronic energy-momentum relation *E*_p_(*k*). Two aspects of this modification are important here.

First, polaron formation is energetically favourable, resulting in the polaronic energy shift *E*_p_(0)≃−*α*_*e−ph*_*ħω*_0_. The effective band-gap for the equilibrium concentration of electron- and hole-polarons is thus reduced: 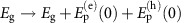. With the estimates above, this reduction largely negates the effect of the exciton binding *E*_X_ and substantially decreases the exponential factor in equation (6). (One could also say that the thermal dissociation of an exciton into a polaron pair is more efficient than into a pair of band carriers.)

**Figure 6:**
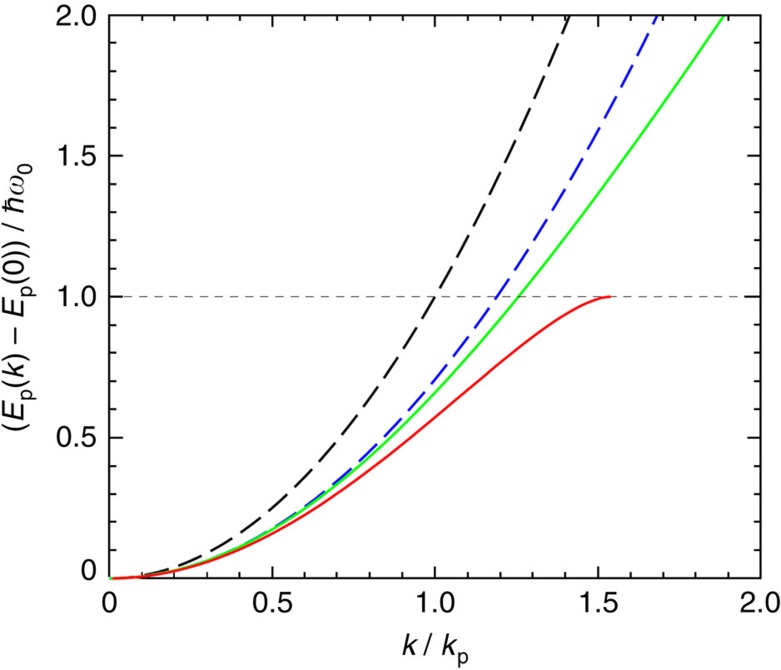
Polaron energy-momentum relation. This plot compares the shape of the energy-momentum curves for different models calculated with the electron–phonon coupling constant *α*_*e−ph*_≃2.5. The energy is measured in units of the vibrational energy *ħω*_0_ and the wave number in units of *k*_p_=(2*mω*_0_/*ħ*)^1/2^. The dashed lines show the parabolic dispersion, for the bare band carrier (black) and for the polaronic carrier with renormalized mass *m*_p_ (blue). The solid lines display the non-parabolic dispersion obtained with variational calculations, according to Lee–Low–Pines theory^11^,^30^ (green curve) and according to Larsen theory^13^,^31^ (red curve). The latter is shown in the limited range of its applicability, but it is this type of the dispersion that was actually confirmed in the state-of-the-art diagrammatic Monte-Carlo calculations^32^.

Second, polarons are ‘heavier' than the band carriers. While this is qualitatively clear already at the level of the effective mass renormalization^12^: *m*→*m*_p_≃*m*(1+*α*_*e−ph*_/6), the actual changes are even more significant, as the polaron dispersion becomes non-parabolic (see Fig. 6 with the accompanying caption, Supplementary Figs 3,4 and Supplementary Note 3)^30^,^31^,^32^. This results in the increased density of the polaronic states and corresponding increase in the equilibrium carrier concentration *n*_eq_. The data in Fig. 6 show that this effect can be substantial: in this illustration, the volume of phase space available for polarons with energies *E*_p_(*k*)−*E*_p_(0)≤*ħω*_0_ increases approximately by a factor of 1.54^3^≃3.7 relative to the bare band carriers (compare the solid red and dashed black lines). Thereby the denominator in equation (3) could additionally increase by about an order of magnitude.

## Discussion

While currently there is no analytical framework that would afford quantitatively reliable calculations of the interplay of excitonic and polaronic effects in the relevant parameter range (all energy parameters: *k*_B_*T*, *ħω*_0_, polaronic and excitonic bindings, are of the same order of magnitude), the above estimates and analysis demonstrate that the polaronic effects might provide an explanation that brings the experimental optical data and our measurements of the radiative recombination coefficient *γ* in a reasonably good agreement with each other. Indeed, the experimental estimates for *γ* derived above from the Hall effect measurements span the range of (1–8) × 10^−11^ cm^3^ s^−1^. On the other hand, the polaronic effects we discussed evidently lead to a substantial decrease of the value in equation (6) evaluated without such effects and likely reducing this estimate below 10^−10^ cm^3^ s^−1^. Given the fact that photo Hall measurements could precisely address only the difference of the hole and electron mobilities, and some uncertainty in theoretical estimates, the consistency of our results appears quite satisfactory. In addition, we note that the non-parabolic polaron dispersion displayed in Fig. 6 clearly features carrier group velocities *v*_gr_(*k*)=∂*E*_p_(*k*)/*ħ*∂*k* considerably lower than those, *ħk/m*, of the bare band carriers. This, of course, leads to lower carrier mobilities as indeed observed in our experiments.

Another important experimental observation is that we have not found the monomolecular decay regime (trapping) down to rather low carrier densities, *n*≈10^15^ cm^−3^ in thin films, and 5 × 10^12^ cm^−3^ in single crystals (Fig. 4). If present, such a regime would manifest itself as a linear σ_PC_(*G*) dependence (*α*=1). The absence of trapping is exactly the reason why bimolecular e-h recombination dominates down to such a low carrier density, leading to a remarkably long carrier lifetime and diffusion length at diluted photo-excitation densities, consistent with prior observations^33^,^34^. In direct-band inorganic semiconductors, even though similarly small values of *γ*=10^−11^—10^−10^ cm^3^ s^−1^ are observed in high-purity crystalline samples, achieving a millisecond carrier lifetime and nearly a millimetre-long diffusion length is unheard of. Ordinarily, other recombination mechanisms such as trapping on recombination centres (or even Auger processes) would start to dominate at a higher crossover carrier density, given the rather small e–h recombination probability. In fact, in well-optimized high-purity single crystals of GaAs, InP or InAs, *τ* and *l* are a few μs and a few tens of μm at best^26^,^27^ (see also http://www.ioffe.ru/SVA/NSM/Semicond/index.html). This accentuates the question of why charge carriers in the (disordered) hybrid perovskites are less affected by trapping. The electronic aspects of the unusual defect physics in CH_3_NH_3_PbI_3_ perovskites have already been discussed^10^. Here, we are wondering if the interaction of defects with methylammonium dipoles could also contribute to the suppression of trapping. Indeed, typical medium-energy traps in semiconductors have energies δ*U*_tr_=0.1–0.3 eV relative to the band edge. Typical physical size of the traps is of the order of a lattice constant, δ*r*_tr_≈5 Å. The local rearrangement of methylammonium dipoles by the effective forces in the vicinity of a defect could then occur, if the potential barrier for the methylammonium dipole rotation, *U*_rot_≈10 meV, is smaller than the energy gain associated with a dipole-trap interaction, for instance, if estimated as: 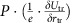
∼10–75 meV (for *P*=2.29 D). The re-arranged dipoles would thus reduce the defect's trapping cross section. Such a defect decoration would be reminiscent of the recently observed trap healing effect at two-dimensional semiconductor/polymer interfaces^35^, with the important distinction that in hybrid perovskites the functional (rotationally responsive) dipoles are naturally available throughout the entire bulk of the sample. The question arises if the dipole rearrangement effect could be greater than that afforded in the standard continuous-dielectric electrostatics. Detailed microscopic computational studies are needed to clarify this issue.

To conclude, we have measured Hall effect in hybrid (organo–inorganic) perovskites films and single crystals and found Hall mobilities ranging from 0.5 to 60 cm^2^ V^−1^ s^−1^, depending on the sample composition and crystallinity. Concurrent measurements of a steady-state photoconductivity and Hall carrier density have allowed to directly determine bimolecular recombination coefficients (as low as 10^−10^ to 10^−11^ cm^3^ s^−1^), carrier lifetimes (up to 30 μs and 2.7 ms in polycrystalline films and single crystals, respectively) and diffusion lengths (up to 23 and 650 μm in films and crystals, respectively). These measurements provide a direct and conclusive evidence of low e–h recombination rates and remarkably weak charge trapping in hybrid perovskites. They show that photophysical properties of these materials are quite different from those of conventional organic or inorganic semiconductors. We emphasize that our study determines these important transport parameters directly, from steady-state transport measurements, relevant to practical applications. The dipolar polaron model has been found helpful to explain the observed relatively low intrinsic carrier mobilities and radiative recombination rates.

## Methods

### Growth of hybrid perovskite thin films and single crystals

For fabrication of thin-film perovskite samples, we followed the published procedures^36^,^37^. Solution of PbI_2_ in dimethylformamide was spin-coated onto cleaned glass substrates, resulting in a homogeneous PbI_2_ films. For solution-grown samples, PbI_2_ films were immersed in a solution of methylammonium iodide in isopropanol. For vapour-grown perovskite films, PbI_2_ was annealed in a saturated methylammonium iodide vapour at 190 °C. The resulting CH_3_NH_3_PbI_3_ thin films were uniform, large-area (centimetre-scale) films on glass. CH_3_NH_3_PbBr_3_ single crystals were grown through anti-vapour diffusion process^38^. In brief, 1:1 ratio of methylammonium bromide (CH_3_NH_3_Br, synthesized following ref. 39) and lead bromide (PbBr_2_, Sigma Aldrich, ⩾98%) were dissolved in *N*,*N*-dimethylformamide. This solution was filtered into an inner container. The inner container was then put into a bigger container with dichloromethane. The outer container was sealed and kept at room temperature. Square bulky CH_3_NH_3_PbBr_3_ single crystals (3–5 mm on a side) grew at the bottom of the inner container within a few days.

### Device fabrication

Devices in Hall bar geometry were fabricated by depositing Au or Ti through a shadow mask on freshly grown films or crystals. After wiring, the devices were capped under vacuum (10^−5^ Torr) with a protective PFPE (perfluoropolyether) oil, which is a chemically and electrically inert perfluorinated polymer. We find that PFPE can effectively protect samples from degradation due to moisture and other environmental factors. Control tests showed that PFPE does not cause any qualitative changes in the electrical properties of hybrid perovskite samples.

### Magneto-transport and photoconductivity measurements

All measurements in this work were carried out at room temperature. a.c. Hall measurements were performed in an a.c. magnetic field of **B**=0.23 T (r.m.s.), referenced to a Stanford Research lock-in amplifier that measures a.c. *V*_Hall_. To reduce the parasitic Faraday induction, frequencies below 1 Hz were typically used. More importantly, *V*_Hall_ generated across the sample at a non-zero d.c. excitation (*I*≠0) was always compared with that generated at zero current (*I*=0), which yields a pure, Faraday-induction-corrected, Hall voltage^15^. We have verified that *V*_Hall_ in all our samples was independent of the frequency in the range 0.3 to 5 Hz, which confirms that undesirable Faraday-induction signal was eliminated. Keithley 6221 current source was used to drive a d.c. excitation current through the sample. Calibration of our a.c. Hall setup has been done by carrying out measurements of a control Si sample with known carrier density and mobility. Longitudinal (photo)conductivity was measured by 4-probe technique, which ensured that ambiguities associated with contact-resistance were eliminated. It is important to emphasize that although 2-probe *σ*_PC_ also exhibits a sublinear power dependence (*σ*_PC_∝*G*^*α*^, with *α*<1), the power exponent *α* in 2-probe measurements may vary in a wider range from sample to sample due to contact effects. In contrast, 4-probe photoconductivity systematically shows *α*=½ at high illumination intensities, which signifies a regime governed by bimolecular recombination. Photo-excitation was achieved by illumination with a calibrated blue LED (max. power: 20 W, *λ*=465 nm) driven by a Keithley 2400 source meter. The highest photon flux used was close to that of one sun (integrating over the part of the spectrum absorbed by the perovskites). Thus, measurements in this work were performed within the range of light intensities relevant for solar cell applications. The errors in Hall mobility values obtained for polycrystalline and single-crystal samples (*μ*_Hall_=8±0.4 and 60±5 cm^2^ V^−1^ s^−1^, respectively) are defined by standard deviation in *V*_Hall_ measurements.

### Data availability

The data that support the findings of this study are available from the corresponding author on request.

## Additional information

**How to cite this article:** Chen, Y. *et al*. Extended carrier lifetimes and diffusion in hybrid perovskites revealed by Hall effect and photoconductivity measurements. *Nat. Commun.* 7:12253 doi: 10.1038/ncomms12253 (2016).

## Supplementary Material

Supplementary Information Supplementary Figures 1-4, Supplementary Notes 1-3 and Supplementary References
