# Supplementary material for: Extended carrier lifetimes and diffusion in hybrid perovskites revealed by Hall effect and photoconductivity measurements
Source: Nat Commun. 2016 Aug 1;7:12253. doi: 10.1038/ncomms12253 (PMC4974647; doi:10.1038/ncomms12253)
Supplement: Supplementary Information — Supplementary Figures 1-4, Supplementary Notes 1-3 and Supplementary References [file ncomms12253-s1.pdf]

SUPPLEMENTARY FIGURES

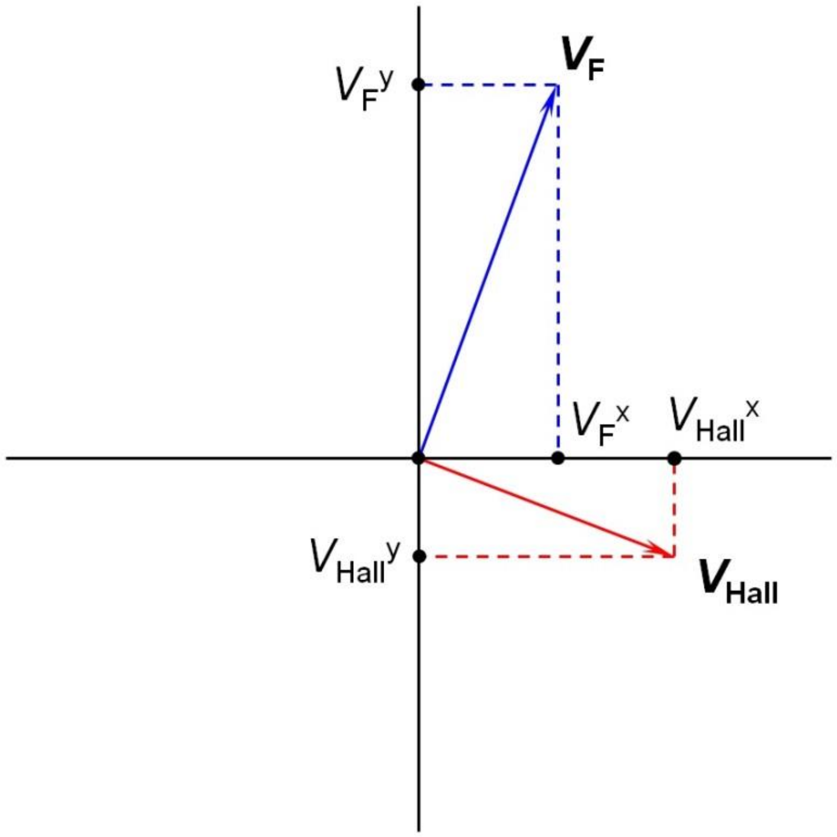

Supplementary Figure 1. A vector representation of the Faraday induction *emf*, $\boldsymbol{V_F}$, and the true Hall voltage, $\boldsymbol{V_{Hall}}$, in the *ac* Hall effect measurements. The measured values in our experiment are the X and Y components of the total voltage, $\boldsymbol{V} = (V^x, V^y)$, presented across the Hall voltage leads of the sample. These components appear as signals on the lock-in amplifier's X and Y channels: $V^x = V_{Hall}^x + V_F^x$, and $V^y = V_{Hall}^y + V_F^y$. When a *dc* longitudinal current is flown through the sample ($I \neq 0$), both $\boldsymbol{V_F}$ and $\boldsymbol{V_{Hall}}$ are generated by the *ac* magnetic field, while for zero excitation ($I = 0$), only $\boldsymbol{V_F}$ is present. Thus, the true Hall voltage defined as $|\boldsymbol{V_{Hall}}| \equiv [(V_{Hall}^x)^2 + (V_{Hall}^y)^2]^{1/2}$ can be found as $|\boldsymbol{V_{Hall}}| \equiv [(V_I^x - V_0^x)^2 + (V_I^y - V_0^y)^2]^{1/2}$, where $\boldsymbol{V_I}$ and $\boldsymbol{V_0}$ are the total voltages across the Hall leads measured at $I \neq 0$ and $I = 0$, respectively.

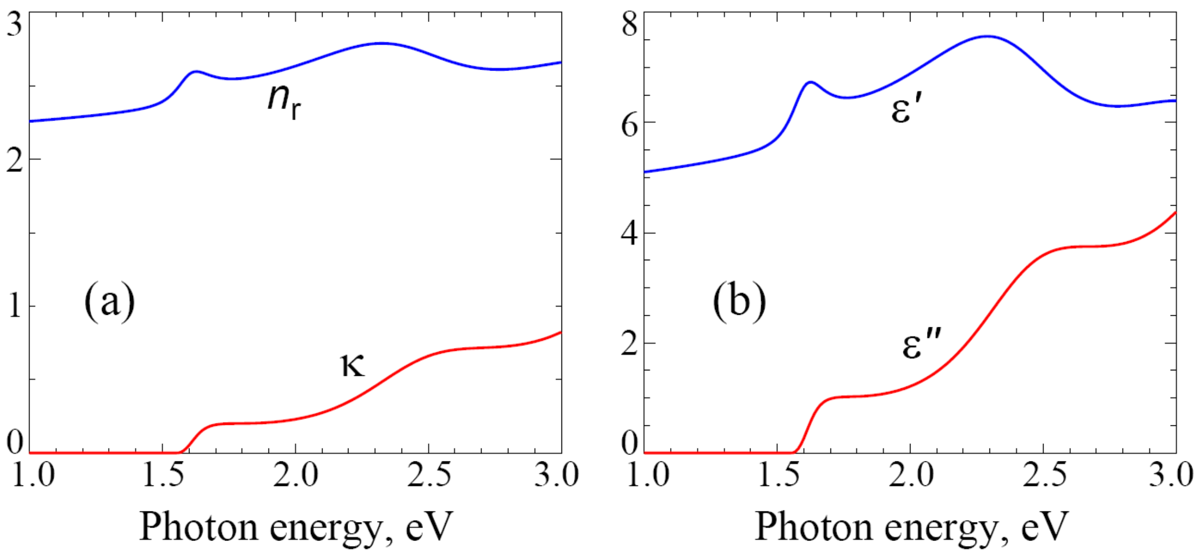

Supplementary Figure 2. Experimental optical parameters of $CH_3NH_3PbI_3$ perovskites as per Ref. [[8]]: (a) in terms of n_r and κ; (b) in terms of the dimensionless dielectric function $\varepsilon = \varepsilon' + i\varepsilon'' = (n_r + i\kappa)^2$.

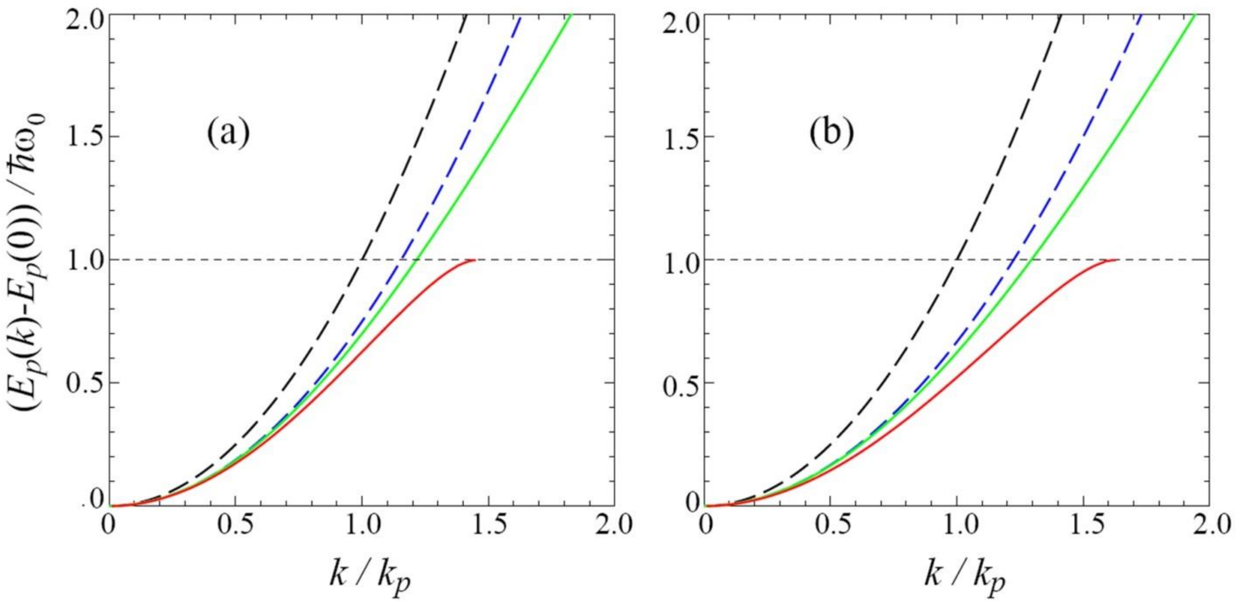

Supplementary Figure 3. Polaron energy-momentum relation. This plot compares the shape of the energy-momentum curves for different models calculated with the electron-phonon coupling constants (a) $\alpha_{\text{e-ph}} = 2.0$ and (b) $\alpha_{\text{e-ph}} = 3.0$. The energy is measured in units of the vibrational energy $\hbar\omega_0$ and the wave number in units of $k_p = (2m\omega_0/\hbar)^{1/2}$. The dashed lines show the parabolic dispersion: black lines for the bare band carrier and blue lines for the polaronic carrier with mass m_p renormalized according to Supplementary Equation (10). The solid lines display the non-parabolic dispersion obtained with variational calculations: according to Lee-Low-Pines theory (green curve) and according to Larsen theory (red).

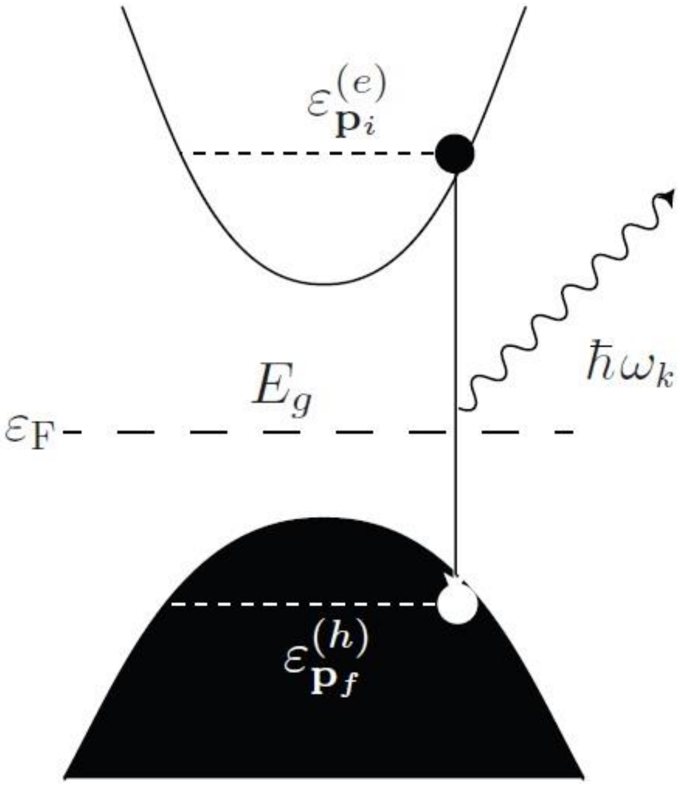

Supplementary Figure 4. The recombination process of electron and hole polarons.

SUPPLEMENTARY NOTES

Supplementary Note 1. On the *ac* Hall effect measurements.

While experimental determination of the fundamental charge transport parameters (e.g., the intrinsic charge carrier mobility, μ) in a steady state may seem to be straightforward, we emphasize that this is not the case in hybrid (organo-inorganic) perovskites for the following reasons. Unambiguous determination of these parameters must rely on precise Hall effect measurements, which are very challenging in highly resistive materials with a relatively low μ, such as organic semiconductors or the hybrid lead-halide perovskites studied here. The major challenges are associated with samples' high resistivity, a negligible (in the dark) density of charge carriers and typically small Hall voltage signals superimposed on a noisy background (for a detailed discussion of similar issues in organic semiconductors see, e.g., [1]). Indeed, by using organic field-effect transistors (OFETs) as a test bed and utilizing the tunable carrier density, n, in OFETs, it has been possible to show that noise in Hall voltage rapidly increases, as the carrier density decreases, as expected for 1/*f*-noise [2]. Therefore, a small intrinsic Hall voltage in low-μ systems can be completely masked by the noise and fluctuations due to scattering and intermittent charge carrier trapping on shallow traps, making it almost impossible to resolve Hall effect even in very high magnetic fields of ~ 10 T. Thus, in order to overcome the problem of low signal-to-noise ratio in low-μ systems, one needs to either develop methods of trap passivation (similar to what we have recently discovered in organic semiconductors [3]) or carry out *ac* Hall measurements with a phase sensitive (lock-in) detection. In addition, it should be pointed out that other artifacts, for instance those related to a parasitic voltages presented across the Hall leads due to the unavoidable misalignment of the Hall voltage probes, thermal contact

emf, as well as Schottky contact resistance, are also frequently overlooked by researchers in Hall effect measurements (for more discussion, see Ref. [1]).

Here, in order to address the problem of signal-to-noise ratio, we have employed an improved *ac* Hall measurement technique [[4]], in which a low frequency (f = 0.5 - 5 Hz) *ac* magnetic field B (*rms* magnitude 0.23 T) is applied perpendicular to the sample's surface, while a *dc* current, I, is passed through the sample. An *ac* Hall voltage, V_{Hall}, is detected across the channel at the same frequency, f, by a phase sensitive lock-in amplifier, which allows to drastically improve the signal-to-noise ratio. Using an *ac* Hall technique is found to be very important for reliable Hall effect measurements in hybrid perovskites. While *dc* Hall measurements do seem to reveal a Hall signal (Fig. 3(d) of the main text), the poor signal-to-noise ratio makes it difficult to obtain quantitative and thus conclusive results. However, even with the much better signal-to-noise ratio in *ac* Hall measurements, one still needs to be extremely careful with certain spurious effects that often lead to data misinterpretation.

One of such artifacts is caused by the *Faraday induction*. The total voltage across the Hall leads, V, consists not only of the true *ac* Hall voltage, V_{Hall}, but also comprises a Faraday induction *emf*, V_{F}, which is a consequence of an *ac* magnetic field passing through a closed-loop circuitry made up with the electric wiring of the sample. This induction signal has the same frequency as the *ac* V_{Hall} and is thus picked up and measured by the same lock-in amplifier. In ideal systems, the Faraday induction signal is phase shifted with respect to the true Hall voltage by $\pi/2$, because the Hall voltage is proportional to B, while the Faraday induction is proportional to $\mathrm{d}B/\mathrm{d}t$, and thus the true Hall signal should present only on the X-channel of the lock-in amplifier, $\boldsymbol{V}_{\mathbf{Hall}} = (V_{\mathrm{Hall}}^{\mathrm{x}}, 0)$, while the Faraday *emf* should only be detected on the Y-channel, $\boldsymbol{V}_{\mathbf{F}} = (0, V_{\mathrm{F}}^{\mathrm{y}})$, making the task of discriminating these signal easier. However, in non-ideal systems,

the phase angle between these signals can somewhat differ from π/2, and the signals themselves can have both of their X and Y components non-zero, $\boldsymbol{V}_{\mathbf{Hall}} = (V_{\mathrm{Hall}}^{\mathrm{x}}, V_{\mathrm{Hall}}^{\mathrm{y}})$ and $\boldsymbol{V}_{\mathbf{F}} = (V_{\mathrm{F}}^{\mathrm{x}}, V_{\mathrm{F}}^{\mathrm{y}})$. This situation is schematically depicted in Supplementary Figure 1. Thus, an additional effort is necessary to extract the true Hall voltage. In principle, the parasitic Faraday induction signal can be much reduced by arranging the sample's wiring in such a way as to minimize the area of the loop and by performing these measurements at a very low frequency (e.g., < 1 Hz). However, even with such an extra care, the Faraday induction may still be comparable or greater than the true *ac* Hall signal, especially in samples with relatively low mobility. In fact, in all the different types of hybrid organo-inorganic perovskites studied here, the Faraday induction *emf* is typically comparable or even greater than the true V_{Hall} signal at the experimental conditions used (that is, rms B = 0.23 T at frequencies in the range f = 0.5 - 5 Hz). This clearly shows that, unless this artifact is carefully addressed, one would mostly measure a Faraday induction signal, rather than the true Hall effect. Fortunately, we find that the Faraday induction signal is rather stable (at a fixed frequency of the magnetic field), and its long-term drift and fluctuations are usually negligible compared to those of the true *ac* Hall voltage. We have always verified this prior to the actual measurements of our samples. Therefore, the Faraday induction voltage, developing between the Hall voltage leads, can be measured as a background occurring at zero source-drain excitation current (that is, the voltage measured between the Hall probes at I = 0). At I = 0, the true *ac* Hall signal must be zero, because there is no longitudinal drift of charge carriers along the channel, and thus any signal detected across the Hall voltage leads is a pure Faraday induction. This induction voltage can then be subtracted from the total signal measured at a finite excitation current, I > 0. For this reason, all our *ac* Hall measurements are performed in a two-step process: first, an *ac* voltage between the Hall leads is measured at I = 0, and then the

measurement is repeated at $I > 0$, followed by the subtraction procedure and other data analysis (Fig. 3(c) of the main text). It is important to mention that because of the non-idealities of the phase angles mentioned above, the subtraction procedure must be performed by treating the Faraday *emf* and the true V_{Hall} as vectors. Thus, the true Hall voltage is obtained as $|\boldsymbol{V}_{\mathbf{Hall}}| \equiv [(V_{\mathrm{Hall}}^{x})^2 + (V_{\mathrm{Hall}}^{y})^2]^{1/2} = [(V_{\mathrm{I}}^{x} - V_0^{x})^2 + (V_{\mathrm{I}}^{y} - V_0^{y})^2]^{1/2}$, where V_{I}^{x} and V_{I}^{y} are the X and Y components of the total voltage measured by the lock-in across the Hall leads when $I \neq 0$, and V_0^{x} and V_0^{y} are the X and Y components of the total voltage measured by the lock-in across the Hall leads when $I = 0$ (Supplementary Figure 1).

<u>Supplementary Note 2.</u> On the extraction of carrier lifetime, τ, and diffusion length, *l*, in the case of thick samples (bulky single crystals).

Our starting point is the rate equation for carrier density n (Eq. 1 in the main text):

$$\frac{dn}{dt} = \kappa G - \frac{n}{\tau} - \gamma n^2 = 0. \qquad (1)$$

For the definition of all the variables and parameters, please see the main text. In the regime dominated by bimolecular recombination, this equation is replaced with:

$$\frac{dn}{dt} = \kappa G - \gamma n^2 = 0\,. \qquad (2)$$

Both the carrier density, n, and the photoexcitation density, G, in this equation are the three-dimensional variables in the units of cm^{-3} and $\mathrm{cm}^{-3}\mathrm{s}^{-1}$, respectively. The Hall carrier density, n_{Hall}, and the incident photon flux, F, experimentally determined in this work are on the contrary the two-dimensional parameters (a projected carrier density and a photon flux incident at the surface) in the units of cm^{-2} and $\mathrm{cm}^{-2}\mathrm{s}^{-1}$, respectively. Thus, these experimental parameters must

be first converted to the three-dimensional variables in order for us to be able to use the above rate equations, Supplementary Equations (1, 2), for the analysis of the data shown in Fig. 4 (main text).

For our $CH_3NH_3PbI_3$ thin film samples, since the film thickness (100 - 200 nm) is comparable to the light penetration length, α^{-1} ~ 80 nm (α is the optical absorption coefficient, not to be confused with the power exponent α in photoconductivity measurements), it is reasonable to use the light penetration length to define the photoexcitation density as: $G = F/\alpha^{-1}$. Similarly, one can define an effective three-dimensional carrier density as: $n = n_{Hall}/\alpha^{-1}$. Using thus obtained G and n, we extract the e-h recombination coefficient γ by fitting the experimental data in Fig. 4 (b, c) of the main text with Supplementary Equation (2). In the regime where a bimolecular recombination dominates (that is, when a square-root power dependence, $\sigma_{PC} \propto G^{1/2}$, is observed), one can define an effective carrier lifetime as: $\tau = (\gamma n)^{-1}$, as explained in the main text, and an effective carrier diffusion length as: $l = (D\tau)^{1/2}$, where D is the diffusion coefficient of the charge carriers related to their mobility, μ, through the Einstein relationship, $D = k_B T\mu/e$. The mobility is obtained independently by performing the Hall effect measurements (see the main text).

For the above analysis of the photocurrent excitation power dependence to be valid, the photoexcitation penetration length α^{-1} must be comparable to the thickness of the sample effectively occupied by photocarriers. This is because in defining the 3D variables, n and G, via the 2D n_{Hall} and F, we have assumed that the volume occupied by photocarriers is comparable to the volume where photon absorption takes place. This assumption is justified in the case of thin-film samples, such as above and in Fig. 4 (b, c) of the main text, whose thickness is ~ α^{-1}, and the carrier diffusion length $l \gg \alpha^{-1}$. In such a case, one can consider that the photoexcitation and

charge carriers occupy the same volume of the sample and are both distributed approximately uniformly through the thickness of the sample, thus allowing to define the effective three-dimensional carrier density as: $n = n_{\mathrm{Hall}}/\alpha^{-1}$.

However, such an analysis becomes invalid in bulk $CH_3NH_3PbBr_3$ single crystals, because their thickness (~ mm) is now much greater than the absorption length of α^{-1} ~ 40 nm for $\lambda = 465$ nm in $CH_3NH_3PbBr_3$, and the photogenerated carriers have room to diffuse deep into the crystal, resulting in a non-uniform carrier distribution with a certain characteristic length, l, much greater than the light penetration length ($l >> \alpha^{-1}$). The precise description of this regime requires a concomitant solution of Supplementary Equation (2) and a diffusion equation for the charge carriers. Nevertheless, the fact that the ½ power dependence is observed in single crystals as well (Fig. 4 (d) of the main text) suggests that despite a relatively short light penetration length α^{-1} ~ 40 nm, the photoexcitation is distributed to a depth comparable to the carrier diffusion length. This can occur for instance via reabsorption. This phenomenon requires further studies, and precise analysis of this in single crystals is beyond the scope of the current work. However, one can still define an effective thickness, d, of the crystal, representing the characteristic depth of the distributions for the photoexcitation and the charge carriers, and define a three-dimensional carrier density as: $n = n_{\mathrm{Hall}}/d$, and a three-dimensional photoexcitation density as: $G = F/d$. Below, we show that this (unknown) thickness d drops out of calculations of the carrier lifetime and diffusion length, and therefore we do not need to know it beforehand. Supplementary Equation (2) can then be rewritten in terms of the experimental (two-dimensional) parameters, n_{Hall} and F, as:

$$\frac{1}{d}\cdot\frac{dn_{Hall}}{dt} = \kappa\cdot\frac{F}{d} - \gamma\left(\frac{n_{Hall}}{d}\right)^2 = 0, \qquad \text{or} \qquad n_{Hall} = \left(\frac{\kappa d}{\gamma}\right)^{1/2}\cdot F^{1/2} \tag{3}$$

By fitting our experimental $n_{Hall}(F)$ dependence (Fig. 4 (d) of the main text) with the above relationship, we experimentally find the slope $\beta \equiv (\kappa d/\gamma)^{1/2}$. We then assume as usual that the photocarrier generation efficiency per photon is 100 % ($\kappa = 1$), which leads to the estimate of the upper bound for γ ($\gamma < d/\beta^2$) and the lower bounds for the photocarrier lifetime, τ, and diffusion length, l, given below. Correspondingly, the effective lifetime of photocarriers can be defined as: $\tau \equiv (\gamma n)^{-1} = \beta^2/(dn) = \beta^2/(n_{Hall})$. Note that this τ is independent of the assumed effective thickness, d, of the sample carrying photogenerated electrons and holes, even though we have introduced d in the first place in order to use Supplementary Equations (1, 2). By applying the above extraction method to the single-crystal data plotted in Fig. 4 (d) of the main text, one obtains the slope $\beta = 2.83\times10^4$ $s^{1/2}cm^{-1}$ and $\tau = 8\times10^8/n_{Hall}$. At the lowest photoexcitation intensity corresponding to $n_{Hall} = 3\times10^{11}$ cm^{-2}, we thus estimate: $\tau = 2.7$ ms, and $l \equiv \sqrt{D\tau} = \sqrt{\frac{\mu_{Hall} k_B T \tau}{e}} = 650$ μm. As argued in the main text, these values represent the lower bounds for the *trap-limited* carrier lifetime, τ_{tr}, and diffusion length, l_{tr}, since the condition $\tau_{tr} \gg \tau = (\gamma n)^{-1}$ must be satisfied in the entire regime dominated by bimolecular recombination (power ½ regime). By using thus estimated carrier diffusion length $l \sim 650$ μm as the effective thickness of the sample populated with photogenerated electrons and holes, we can also explicitly estimate the *e-h* recombination coefficient: $\gamma = d/\beta^2 \sim l/\beta^2 = 8\times10^{-11}$ cm^3s^{-1}.

The much greater τ and l extracted above in $CH_3NH_3PbBr_3$ single crystals appear to be mainly the result of a much thicker layer of the sample populated with photocarriers upon photoexcitation (afforded simply by the macroscopic thickness of these crystals), which leads to a significantly lower 3D carrier density than in thin films. Indeed, the lowest photoexcitation intensity in the single-crystal data in Fig. 4 (d) of the main text corresponds to $n_{Hall} = 3\times10^{11}$ cm^{-2}

and $n \sim n_{Hall}/l$ = 4.6×10^{12} cm^{-3}, which is almost two and a half orders of magnitude lower than the carrier density in our thin-film devices (~ 10^{15} cm^{-3}) at a similar photoexcitation intensity. In thin-film samples, all the photogenerated carriers are confined to the small physical thickness of the film, resulting in a high carrier density. In such a case, since the *e-h* recombination lifetime is inversely proportional to the carrier density $\tau = (\gamma n)^{-1}$, electrons and holes have much higher probability to recombine. Again, we emphasize that in the bimolecular recombination regime, both τ and l depend on the carrier density n, and thus these parameters must always be mentioned with a reference to a particular carrier density.

Supplementary Note 3. van Roosbroeck-Shockley theory of radiative recombination and polaronic effects.

3.1. Radiative recombination rate and the experimental optical data.

W. van Roosbroeck and W. Shockley (vRS) have developed the general formalism, based on the principle of detailed balance, which was originally used to calculate radiative recombination rates in Ge [5] and then successfully applied to a variety of other inorganic semiconductors (see a review by Y. P. Varshni [6] and a textbook description in Ref. [7]). In their approach, the radiative electron-hole recombination coefficient γ is established by the system properties in the thermal equilibrium (that is, in the dark):

$$\gamma = R_{eq}/n_{eq}^2, \quad (4)$$

where R_{eq} and n_{eq} are the equilibrium (dark) recombination rate and concentration of electrons (equal to that of holes), respectively. Furthermore, R_{eq} is related thermodynamically to the optical properties of the system as [7]:

$$R_{eq} = \frac{2}{\pi^2 c^3} \int_0^\infty \frac{n_r^2\, \kappa\, \omega^3\, d\omega}{\exp(\hbar\omega/k_B T) - 1}\,. \tag{5}$$

Here c is the speed of light in vacuum, and the optical properties are described by the frequency ω-dependent refraction, $n_r(\omega)$, and extinction, $\kappa(\omega)$, coefficients. An advantage of Supplementary Equation (5) is that it relates the rate to directly measurable experimental quantities and can be evaluated as such. For these calculations, we use here the accurate parameterized description [8] of optical parameters of $CH_3NH_3PbI_3$ perovskites derived from their ellipsometric measurements, which are displayed in Supplementary Figure 2 for reference. In this parameterization, the onset of optical absorption ($\kappa > 0$) takes place at $E_{opt} = 1.553$ eV. On the other hand, there is no direct measurement of the equilibrium (dark) concentration n_{eq}. We will explore below the influence of the interaction of conventional band carriers with the MA dipole reorganization ("dipolar phonons") on this quantity.

3.2. The electron-phonon coupling constant.

In the theory of polarons [9,10,11], it is common to define the dimensionless coupling constant between a band charge carrier (effective mass m) and the polar medium as:

$$\alpha_{\text{e-ph}} = \frac{1}{2}\left(\frac{1}{\varepsilon_\infty} - \frac{1}{\varepsilon_s}\right)\frac{k_e e^2}{\hbar\omega_0}\left(\frac{2m\omega_0}{\hbar}\right)^{1/2}. \tag{6}$$

Here, ε_s and ε_∞ are the static and high-frequency (optical) dielectric constants, respectively, k_e the Coulomb's law constant, and ω_0 the frequency of the longitudinal dipolar vibrational (phonon) mode. To make numerical estimates, from a range of published dielectric constants, $\varepsilon_s \approx 18$-36, $\varepsilon_\infty \sim 5$-$6$ [8,12], we will specify here $\varepsilon_s = 25$ and $\varepsilon_\infty = 5$ (see Supplementary Figure 2 (b)), along with the effective band mass $m = 0.2m_0$, where m_0 is the free electron mass [13]. The longitudinal phonon frequency $\omega_0 = (\varepsilon_s/\varepsilon_\infty)^{1/2}\omega_t$ is known [10] to relate to the ordinary

(transverse) vibrational frequency ω_t. We estimate the latter by equating the kinetic energy of the dipole oscillations to the potential energy of the nearest-neighbor dipole interaction as $\frac{I\omega_t^2}{2} = \frac{\kappa_e d^2}{\varepsilon_\infty a^3}$, where d is the dipole moment, $d \approx 2.3$ D [14]. The moment of inertia is further estimated from the energy barrier for rotation of the methyl-ammonium (MA) dipole in the perovskite cage: $E_r = 1.3\times10^3$ J/mol [14]. With the cage size $a \approx 6.3$ Å, $\omega_t = \sqrt{\frac{\kappa_e d^2}{\varepsilon_\infty a^3} E_r} \approx 8\times10^{12}$ s^{-1}, thus leading to $\hbar\omega_0 \approx 11.8$ meV and, by Supplementary Equation (6), to $\alpha_{\text{e-ph}} \approx 2.4$. In the parlance of the polaron theory [9,10,11], such magnitudes of the electron-phonon interaction constant are classified as intermediate coupling (as compared to weak coupling at $\alpha_{\text{e-ph}} < 1$ and strong coupling at $\alpha_{\text{e-ph}}$ comparable to or larger than 10). The last factor in Supplementary Equation (6) defines a characteristic polaronic wave number $k_p = (2m\omega_0/\hbar)^{1/2}$ and, correspondingly, a characteristic polaronic length scale $1/k_p$ [10], yielding about 40Å for our estimates.

3.3. The polaron energy spectrum.

As a result of the dressing by the phonon cloud [10], the standard energy-momentum relation for the band carriers,

$$E(k) = \hbar^2 k^2/2m, \tag{7}$$

gets replaced by the polaronic relation $E_p(k)$. This transformation is particularly clear at small momenta, $k \ll k_p$, when

$$E_p(k) \simeq E_p(0) + \hbar^2 k^2/2m_p \tag{8}$$

corresponds just to the appearance of the polaronic energy shift and the renormalized (heavier) polaronic effective mass, m_{p}. For the intermediate-coupling polarons, these are well approximated by the known [10] expressions

$$E_p(0) \simeq -\alpha_{\text{e-ph}}\hbar\omega_0 \tag{9}$$

and

$$m_p \simeq m(1+\alpha_{\text{e-ph}}/6). \tag{10}$$

At larger momenta, however, the modification of the polaronic spectrum is more significant than would be suggested just by mass renormalization (8), (9) – the energy-momentum relation becomes *non-parabolic*. Various variational approaches were developed to explore this issue, such as the Lee-Low-Pines [9,15] and Larsen [11,16] theories. We refer the reader to those citations for analytical details. Here we provide graphical illustrations by calculating the resulting dispersion curves for a few values of the electron-phonon coupling constant, $\alpha_{\text{e-ph}}$ = 2.0, 3.0 (displayed in Supplementary Figure 3), and 2.5 (displayed in the main text, Fig. 6). The non-parabolic relations clearly exhibit densities of polaronic states substantially increased in comparison with the bare band carriers. The increased density of states would be contributing to greater values of the equilibrium polaron concentration n_{eq} in the denominator of Supplementary Equation (4), thereby decreasing the radiative recombination coefficient γ for the given (fixed) experimental optical parameters in Supplementary Equation (5). It should be emphasized that while Larsen theory (red curves in Supplementary Figure 3) is applicable only for a limited range of polaron energies: $E_p(k) - E_p(0) \le \hbar\omega_0$, it is actually this type of the polaron dispersion that was confirmed in the modern state-of-the-art diagrammatic Monte-Carlo calculations [17]. The numerical magnitudes of the increased volume of the phase space in this limited region of

polaron energies is worth mentioning: in Supplementary Figure 3 (a), it is $1.45^3 \approx 3.05$, and in Supplementary Figure 3 (b) it is $1.63^3 \approx 4.33$, quite substantial factors for contributions to n_{eq}.

Non-parabolic dispersion curves in Supplementary Figure 3, of course, also exhibit polaron group velocities lower than would be suggested just by the renormalized mass (10). In fact, in Larsen theory, the group velocities vanish upon the approach of the momenta $\hbar k$ to their critical values (see discussions of this point in Refs. [10,17]). Lower group velocities would correspondingly result in lower carrier mobilities.

3.4. The application of vRS theory to the polaronic bands in the parabolic approximation.

It is also instructive to evaluate the radiative recombination rate within the confines of the standard theory of electron-hole recombination in semiconductors (see, e.g., [[6]] and references therein) under the only assumption of the renormalization of the parabolic band parameters, that is, if the relaxed charge carriers become polarons with some effective masses. The effective polaron masses themselves could then be estimated based on the experimental mobility and Drude model and the calculated recombination coefficient (4) compared with the experimentally obtained. The polaronic energy shifts here are assumed already included in the renormalized gap energy E_g. For clarity, we will take the band mass of electrons and holes approximately equal to each other: $m_e \simeq m_h = m$. As a result, the effective masses of electron and hole polarons would be equal to m_p.

Supplementary Figure 4 shows the schematics of the recombination process, to which we apply the standard [[6]] calculation procedure. The recombination time, τ, can be computed via the usual Fermi golden rule expression:

$$\frac{1}{\tau(\boldsymbol{p_i})} = \frac{2\pi}{\hbar}\int \frac{d^3p_f}{(2\pi\hbar)^3}\frac{d^3k}{(2\pi)^3}\left|M_{fi}\right|^2(2\pi\hbar)^3\delta\big(\boldsymbol{p}_f + \hbar\boldsymbol{k} - \boldsymbol{p_i}\big)\,\delta\left(\varepsilon^{(h)}_{\boldsymbol{p}_f} + \hbar\omega_k - \varepsilon^{(e)}_{\boldsymbol{p}_i}\right), \quad (11)$$

where M_{fi} is the transition (dipole) matrix element:

$$M_{fi} = e\sqrt{2\pi k_e\hbar\omega}\langle f|\boldsymbol{n}_k \times \boldsymbol{r}|i\rangle, \quad (12)$$

and $\varepsilon^{(h)}_{\boldsymbol{p}_f}, \varepsilon^{(e)}_{\boldsymbol{p}_i}$, $\hbar\omega_k = c\hbar k$ are the energies of the final and initial polaron, and of the photon states, respectively. The unit vector is $\mathbf{n}_k = \mathbf{k}/k$. The parabolic dispersions for the electron and hole polarons read:

$$\varepsilon^{(h)}_{\boldsymbol{p}} = -\frac{\boldsymbol{p}^2}{2m_p},\ \varepsilon^{(e)}_{\boldsymbol{p}} = \frac{\boldsymbol{p}^2}{2m_p} + E_g. \quad (13)$$

The integral in Supplementary Equation (11) is taken over all possible final states of the irradiated photon and the possible states of the hole. The total equilibrium unit-volume recombination rate, R_{eq}, reads:

$$R_{eq} = \int \frac{d^3p_i}{(2\pi\hbar)^3}\frac{1}{\tau(\boldsymbol{p_i})} f\left(\varepsilon^{(e)}_{\boldsymbol{p_i}}\right)\left[\left(1 - f\left(\varepsilon^{(h)}_{\boldsymbol{p_f}}\right)\right)\right], \quad (14)$$

where the factor $f\left(\varepsilon^{(e)}_{\boldsymbol{p}_i}\right)\left[\left(1 - f\left(\varepsilon^{(h)}_{\boldsymbol{p_f}}\right)\right)\right]$ corresponds to the averaging with thermal distribution functions over all possible initial states of the electron and hole polarons.

The calculations are particularly easy to perform for the equilibrium (dark) case. It is known (see also Supplementary Equation (4)) that the non-equilibrium (that is, under photoexcitation) recombination rate, $R_{non\text{-}eq}$, is related to the equilibrium rate, R_{eq}, as follows:

$$R_{non-eq} = R_{eq}\frac{np}{n_{eq}^2}, \quad (15)$$

where n and p are the non-equilibrium (photoexcited) concentrations of electrons and holes (for which we assume, $n \approx p$), and n_{eq} or p_{eq} ($n_{eq} = p_{eq}$) are the equilibrium (dark) concentrations of electrons and holes. The average effective recombination time is then defined as:

$$\frac{1}{\tau} = \frac{R_{non-eq}}{n} \tag{16}$$

while $\boldsymbol{\gamma} \equiv \frac{\boldsymbol{R_{eq}}}{\boldsymbol{n_{eq}^2}} = \frac{\boldsymbol{R_{non-eq}}}{\boldsymbol{np}}$.

3.5. The computation of recombination rate R in hybrid perovskites.

The integral over the final states d^3p_f in Supplementary Equation (11) trivially removes the momentum δ-function and sets $\mathbf{p}_f = \mathbf{p}_i - \hbar\mathbf{k}$, while the integral over the initial states d^3p_i in Supplementary Equation (14) removes the energy δ-function. The important simplification comes from the fact that the characteristic electron and hole momentum (wave-vector) is of the order of:

$$k_e \sim k_h \sim \frac{1}{\hbar}\sqrt{2m_p k_B T} > \frac{1}{\hbar}\sqrt{2m^* k_B T} \sim 2\times10^8 \text{ m}^{-1}, \tag{17}$$

while the characteristic photon wave-vector is:

$$k_{ph} = \frac{E_g}{c\hbar} \sim 10^7 \text{ m}^{-1} \tag{18}$$

Thus, for our calculation $k_{ph} \ll k_e, k_h$. Therefore, when dealing with energy δ-function, we may set $\varepsilon^{(e)}_{\boldsymbol{p_i}-\boldsymbol{k}} \approx \varepsilon^{(e)}_{\boldsymbol{p_i}}$. Rewriting the δ-function as $\delta\left(c\hbar k - \frac{p^2}{m_p} - E_g\right)$, we integrate over $p_i^2 dp_i$ to obtain the so-called joint density of states factor $\frac{1}{2\pi^2\hbar^3}\sqrt{(c\hbar k - E_g)}\cdot {m_p}^{3/2}/2$. The matrix

element $|M_{fi}|^2$ is to be averaged over the direction of the optical emission and summed over possible photon polarizations:

$$\langle \left| M_{fi} \right|^2 \rangle_\Omega = \frac{8\pi}{3} k_e e^2 \hbar\omega \langle f|\boldsymbol{r}|i\rangle^2 \tag{19}$$

Taking into account the position of the Fermi level (Supplementary Figure 4), we set $f\left(\varepsilon_{\boldsymbol{p}}^{(h)}\right) \approx 1$ and $f\left(\varepsilon_{\boldsymbol{p}}^{(e)}\right) \approx 0$. As a result, in the limit $E_g \gg k_B T$, one gets the following expression for the recombination rate:

$$R_{eq} = \frac{4k_e e^2 c m_p^{3/2}}{3\hbar^3} \int_{E_g/c\hbar}^{\infty} \frac{4\pi k^3 dk}{(2\pi)^3} \frac{|\langle f|\boldsymbol{r}|i\rangle|^2 \sqrt{(c\hbar k - E_g)}}{e^{c\hbar k/k_B T} - 1} \tag{20}$$

The integral (20) can be taken analytically, given the simplification provided by the condition $E_g \gg k_B T$. The dipole matrix element is expressed via the so-called Kane energy, E_P:

$$\langle f|\boldsymbol{r}|i\rangle^2 = \frac{\hbar^2 E_P}{2E_g^2 m} \tag{21}$$

Kane energy E_P in hybrid perovskites has been estimated in numerical simulations of the optical absorption of these materials [[18],[19]]:

$$E_P = 6.3 \text{ eV} \tag{22}$$

Combining Eqs. (15), (16) and the identity for the equilibrium carrier concentration n_{eq}:

$$n_{eq}^2 = 4\frac{(k_B T m_p)^3}{(2\pi)^3 \hbar^6} e^{-\frac{E_g}{k_B T}} \tag{23}$$

one arrives at the final expression for the carrier recombination time τ as a function of photoexcited carrier density, *p*, and polaron effective mass, m_p:

$$\tau = \frac{3}{2\sqrt{\pi}} \frac{c^3\, m\, m_p^{3/2}}{p k_e e^2 \hbar E_g E_P} (k_B T)^{3/2} \tag{24}$$

3.6. Estimates of the recombination time τ and the bimolecular recombination coefficient γ.

As discussed at the setup, here our goal is not to calculate the polaron mass but to estimate it from the experimental data for mobility. Such an estimate for the polaron effective mass in perovskites can be obtained using the following qualitative arguments. The carrier mobility of the material can be expressed as follows:

$$\mu = \frac{el}{m_p\, v}, \quad v \sim \sqrt{\frac{k_B T}{m_p}}, \tag{25}$$

where l is the carrier momentum relaxation length, and v the thermal velocity. Within the polaron picture we discuss, it is clear that l should be at least of the order of one lattice constant: $l > a$. Otherwise, the transport would be of the hopping type that is appropriate for a very different type of so-called small-radius polarons.[10] With this limitation on l,

$$m_p > \frac{e^2 a^2}{\mu^2 k_B T} \approx 3m, \tag{26}$$

where for the carrier mobility we took the value in the middle of the range experimentally revealed in our Hall measurements, $\mu \sim 30$ cm^{2}V^{-1}s^{-1} (see main text). Hence, following Supplementary Equation (24) above, we extract the following *e-h* recombination time in our samples (at the lowest photoexcitation intensity used in our experiment):

$$\tau \; > \; 1 \text{ ms.} \tag{27}$$

In this computation, we used p = 4.6×10^{12} cm^{-3} for the carrier concentration from the experiment (see main text), for the band mass $m = 0.15m_0$, and for the band gap E_g = 1.6 eV. The bimolecular recombination coefficient γ can also be estimated from Supplementary Equation (24):

$$\gamma \equiv (\tau p)^{-1} < 2\times10^{-10} \text{ cm}^3\text{s}^{-1} \tag{28}$$

The resulting magnitudes (28) of the bimolecular recombination coefficient γ (and recombination time τ (27) for a specific carrier density) thus appear in a reasonably good agreement with the experiment.

SUPPLEMENTARY REFERENCES

1. Podzorov, V. Organic single crystals: addressing the fundamentals of organic electronics, *MRS Bulletin* **38**, 15-24 (2013).

2. Podzorov, V., Menard, E., Rogers, J. A. & Gershenson, M. E. Hall effect in the accumulation layers on the surface of organic semiconductors, *Phys. Rev. Lett.* **95**, 226601 (2005).

3. Lee, B., Chen, Y., Fu, D., Yi, H. T., Czelen, K., Najafov, H. & Podzorov, V. Trap healing and ultralow-noise Hall effect at the surface of organic semiconductors, *Nature Mater.* **12**, 1125-1129 (2013).

4. Chen, Y., Yi, H. T. & Podzorov, V. High resolution *ac* measurements of the Hall effect in organic field-effect transistors, *Phys. Rev. Applied* **5**, 034008 (2016).

5. van Roosbroeck, W. & Shockley, W. Photon-Radiative Recombination of Electrons and Holes in Germanium, *Phys. Rev*. **94**, 1558 (1954).

6. Varshni, Y. P. Band-to-band radiative recombination in groups IV, VI and III-V semiconductors (I). *Physica Status Solidi* **19**, 459 (1967).

7. Pankove, J. I. *Optical Processes in Semiconductors* (New York, Dover Publications, 1971).

8. Löper, P. *et al.*, Complex Refractive Index Spectra of $CH_3NH_3PbI_3$ Perovskite Thin Films Determined by Spectroscopic Ellipsometry and Spectrophotometry, *J. Phys. Chem. Lett.* **6**, 66-71 (2015).

9. Kuper, C. G. & Whitfield, G. D. (eds) *Polarons and Excitons* (New York, Plenum Press, 1963).

10. Appel, J. *Polarons*, in *Solid State Physics*, Vol. 21 (eds F. Seitz *et al.*), 193 - 391 (New York, Academic Press, 1968).

11. Devreese, J. T. (ed) *Polarons in Ionic Crystals and Polar Semiconductors* (Amsterdam, North-Holland, 1972).

12. Brivio, F., Walker, A. B. & Walsh, A. Structural and electronic properties of hybrid perovskites for high-efficiency thin-film photovoltaics from first-principles. *APL Mater*. **1**, 042111 (2013).

13. Giorgi, G., Fujisawa, J.-I., Segawa, H. & Yamashita, K. Small Photocarrier Effective Masses Featuring Ambipolar Transport in Methylammonium Lead Iodide Perovskite: A Density Functional Analysis, *J. Phys. Chem. Lett.* **4**(24), 42134216 (2013).

14. Frost, J. M., Butler, K. T., Brivio, F., Hendon, C. H., van Schilfgaarde, M. & Walsh, A. Atomistic origins of high-performance in hybrid halide perovskite solar cells. *Nano Lett*. **14** (5), 2584 - 2590 (2014).

15. Lee, T. D., Low F. E. & Pines, D. The Motion of Slow Electrons in a Polar Crystal, *Phys. Rev.* **90**, 297 (1953).

16. Larsen, D. M. Polaron Energy Spectrum, *Phys. Rev.* **144**, 697-702 (1966).

17. Prokof'ev, N. V. & Svistunov, B. S. Polaron Problem by Diagrammatic Quantum Monte Carlo, *Phys. Rev. Lett.* **81**, 2514-2517 (1998).

18. Even, J., Pedesseau, L. & Katan, C. Analysis of multivalley and multibandgap absorption and enhancement of free carriers related to exciton screening in hybrid perovskites. *J. Phys. Chem.* C, **118**, 11566 (2014).

19. Fang, H.-H., Raissa, R., Abdu-Aguye, M., Adjokatse, S., Blake, G. R., Even, J. & Loi, M. A. Photophysics of organic-inorganic hybrid lead iodide perovskite single crystals, *Adv. Funct. Mater*. **25**, 23782385 (2015).
